# Free flap reconstruction of scalp in a case of advanced lung cancer with brain metastasis: A case report

**DOI:** 10.1097/MD.0000000000035097

**Published:** 2023-09-08

**Authors:** Dong Yun Lee, SooA Lim, SuRak Eo, Jung Soo Yoon

**Affiliations:** a Department of Plastic and Reconstructive Surgery, DongGuk University Medical Center, GoYang, South Korea.

**Keywords:** advanced cancer, free tissue transfer, lung cancer, metastasis

## Abstract

**Rationale:**

Reconstruction of wound complications in patients with advanced cancer with distant metastases is challenging for plastic surgeons. This may be due to the cancer patients’ hypercoagulability and potential intolerance to general anesthesia. This article aimed to discuss the risk of free-flap reconstruction in such cases.

**Patient concerns:**

The patient was a 58-year-old female with advanced non-small cell lung cancer and brain metastasis. The patient underwent brain radiotherapy and chemotherapy through the Ommaya Reservoir.

**Diagnoses:**

A year ago, she underwent several local flap closures for recurrent wound healing failure due to wound complications, including infection, wound dehiscence, and subsequent device removal.

**Interventions:**

A radial forearm free flap was created under general anesthesia. The patient was discharged in the third postoperative week since the flap remained stable.

**Outcomes:**

At follow-up a month thereafter, the patient exhibited signs of recovery without any complications even while continuing her chemotherapeutic regimen.

**Lessons:**

Free flap placement is not an absolute contraindication in cancer patients with distant metastases. Nevertheless, it is associated with clinical challenges and operator hesitancy. This is a case of a successful free flap in a cancer patient with hypercoagulability and suspected floating tumor cells. Postoperative management, in this case, is appropriate.

## 1. Introduction

Patients with advanced cancer and distant metastases often experience various wound complications resulting in wound healing failure. Distant metastases indicate lymphovascular infiltration of the tumor cells, and those tumor cells themselves interfere with vascularization, which interferes with wound healing.^[1]^ Moreover, palliative chemotherapy is associated with delays in wound healing. This delay can be attributed to the pharmacological effects of chemotherapeutic agents, which impede the normal cellular processes involved in wound healing. The cytotoxic properties of these agents affect cell proliferation, migration, and collagen synthesis, all of which play crucial roles in the timely and effective healing of wounds.^[2]^ In addition, cancer patients have hypercoagulability,^[3,4]^ which affects flap stabilization. Free-flap procedures require adequate blood flow.^[5,6]^ However, in cancer patients, aforementioned conditions increase the risk of complications that would result in hesitation on the surgeon’s end.

Although there are no documented contraindications for the free-flap procedure in patients with cancer with distant metastases,^[7,8]^ it poses clinical challenges; however, there are only indications in elderly patients. Furthermore, there are no established contraindications for late-stage cancer.^[7–10]^

In this case report, we encountered a patient with advanced lung cancer and brain metastasis who experienced wound healing failure. After a thorough evaluation of her risk factors, including hypercoagulable state, general condition, and survival expectancy, we performed free-flap reconstruction. Free flaps allow complete wound healing and even enable continuity of chemotherapy.

## 2. Case presentation

A 58-year-old female patient was referred to our plastic and reconstructive surgery department with recurrent wound healing failure on her scalp. She was diagnosed with end-stage non-small cell lung cancer with brain metastasis in 2014 (stage T1bN0M1b); thereafter, she underwent 2 rounds of brain radiotherapy in her right temporal region and right basal ganglia. Moreover, she had a subsequent chemotherapy with an Ommaya reservoir and an intratumoral catheter in her left frontal region due to radiation necrosis with cystic change. However, due to repeated infections, meningitis, and wound dehiscence, the Ommaya Reservoir was removed in November 2021. Due to delayed wound healing and recurrent dehiscence with bone exposure at the site of the Ommaya procedure, a local rotation flap was performed in January 2022 and another rotation flap with Megaderm bone closure was performed in July 2022. She was treated with long-term oral ciprofloxacin for the subcutaneous infection. She was then referred to our department for the management of chronic wounds in November 2022. She never smoked. Additionally, she had no underlying medical conditions other than prolonged PT INR. The lung cancer had invaded her left upper lung, with no distant metastases other than to the brain.

**Figure 1.**
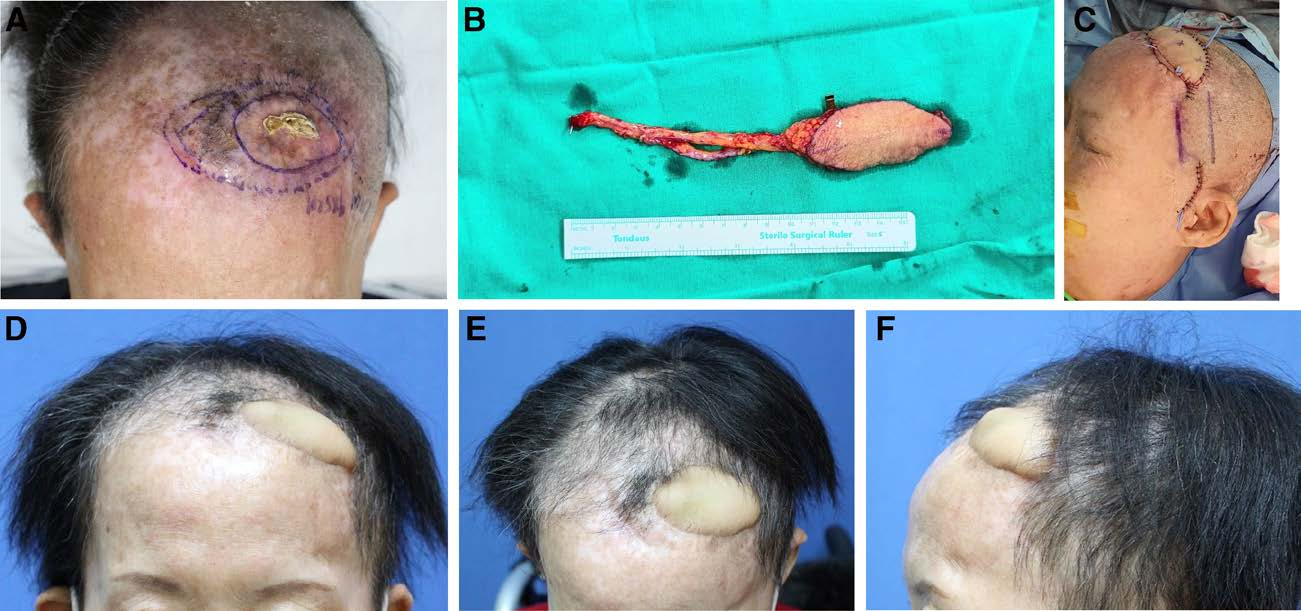
Free flap reconstruction in a 58-year-old female with chronic wound on the frontal scalp. A: Preoperative view; B: Intraoperative view. A left radial forearm free flap was harvested; C: Radial forearm free flap inset for coverage of the frontal scalp defect; D: Postoperative 6-month view (anterior view); E: Postoperative 6-month view (bird’s eye view); F: Postoperative 6-month view (lateral view) showing the decreased bulkiness of the flap. The wound recovered without any surgical complications. No wound complications were identified despite the ongoing chemotherapy.

An infected non-healing wound occurred in the left frontal area. There was a wide scarred area with a necrotic yellow eschar in the center due to multiple radiotherapy sessions and repeated local flap surgeries due to wound complications (Fig. 1A). After wide resection of the unviable tissue and burring of the outer table of the skull, through which the Ommaya Reservoir had been inserted, fresh bleeding was observed. The final defect size was 9 × 5 cm. A radial forearm free flap was carefully planned considering 3 risks: the risk of general anesthesia due to lung cancer, the possibility of flap failure due to delayed wound healing, and the patient’s hypercoagulable state due to cancer. The donor site was harvested from the left forearm (10 × 5 cm). The radial forearm free flap pedicle was the radial artery and venae comitant, and the pedicle length was 11 cm (Fig. 1B). The superficial temporal artery and vein were selected as recipient vessels. Microvascular anastomoses of the arteries and veins were performed using the end-to-end technique. The surgeries were uneventfully completed (Fig. 1C).

After surgery, blood flow was checked using Doppler every 3 hours, while flap viability was continuously checked visually. Eglandin was intravenously administered during the first postoperative week to prevent hypercoagulability. Unlike the routine postoperative regimen for free flaps in our center, 5000 IU per day of intravenous heparin was additionally administered during the first postoperative week. On the second postoperative day, bleeding was observed during dressing and flap monitoring; hence, electrocoagulation was performed under local anesthesia.

At the 6-month postoperative follow-up, the patient had no special complications (Fig. 1D–F). Healing was successful. The patient was also able to tolerate chemotherapy, which was resumed at the second postoperative month.

## 3. Discussion and conclusions

In most cases, large tissue defects occur after extensive tumor resection in patients with head and neck malignancies.^[8,9]^ These defects can be reconstructed using several methods, including skin grafts, local flaps, distant flaps, and free flaps. Free tissue transfers were first introduced clinically by Daniel and Taylor in 1973.^[11]^ With continuous research into donor selection and techniques, free flaps are now the gold standard for head and neck reconstruction. Free flaps are widely used because they can be reconstructed in a single operation, they have a wide selection of flaps, they can possibly be used for combined grafting for functional reconstruction, and they allow 2 surgical teams to operate simultaneously.^[10]^ In addition, a free flap with sufficient volume of soft tissue is required for complete wound healing. However, free-flap surgery is highly technical and longer. Moreover, its success depends on the patient’s vascular status and systemic condition. Known contraindications for the use of free flaps include severe peripheral vascular disease,^[5]^ lack of adequate host vessel availability, medical instability,^[12]^ and the need for vasopressors.^[13]^

Several pre-operative factors contribute to failure of free-flap surgery. The first is hypercoagulability.^[3,4]^ Flap loss most often occurs because of thrombosis of the microvascular pedicle secondary to technical factors, such as intimal damage, vessel kinking, and technical errors regarding the anastomosis. However, despite appropriate surgical techniques and follow-up, flap failure can occur due to systemic influences. There is considerable evidence that thrombosis is a common complication of malignancy and the second most frequent cause of death in cancer patients.^[3]^ Prothrombotic factors in cancer include procoagulant/fibrinolytic substances and inflammatory cytokines secreted by the tumor and the physical interaction between tumor cells and blood (monocytes, platelets, neutrophils) or vascular cells. Other mechanisms of thrombus promotion in malignancy include nonspecific factors, such as generation of acute-phase reactants, necrosis (inflammation), abnormal protein metabolism (paraproteinemia), and hemodynamic compromise (stasis). In addition, anticancer therapy (surgery/chemotherapy/hormone therapy) may significantly increase the risk of thromboembolic events via stimulation of procoagulant release and tissue factor production of host cells or endothelial damage.^[2]^ In addition, floating malignant cells attached to the vessel wall in patients with distant metastases may play a major role in promoting localized clotting activation and thrombus formation through the release of cytokines and subsequent adhesion of other cells, including leukocytes and platelets. The adhesion of tumor cells to each other or to vascular cells may also facilitate cell migration and extravasation. With the expectation of hypercoagulability in our patient, 5000 IU per day of IV heparin was additionally administered for the first postoperative week to control the risk of thrombotic events. A single agent of intravenous prostaglandin E1 was administered for a week as a routine postoperative regimen in free flap cases in our center.

The second factors were advanced age and comorbidities.^[7–10]^ Although advanced age is not a contraindication for free flaps according to articles by Sorg et al^[7]^ and Parsemain et al,^[10]^ it is associated with surgical failure in several studies, including Fagin et al^[9]^ In this case, the patient was under the age of 60 years, which was not deemed elderly. However, our patient had advanced lung cancer; therefore, tolerability to general anesthesia was a significant concern. Thorough preoperative assessment of pulmonary function, cardiac status, and other comorbidities is necessary to determine the suitability of patients for surgery. Close monitoring during the perioperative period and careful titration of the anesthetic agents can help minimize the risk of complications.

The third factor to consider is prolonged operation time. Patients with advanced cancer often have comorbidities that can lengthen the operation time.^[3]^ It is therefore crucial to meticulously plan and optimize the surgical approach to minimize the operative time. Close collaboration with a multidisciplinary team, including medical oncologists and anesthesiologists, is essential to ensure that the patient’s general condition is suitable for the procedure. In particular, a surgery exceeding 8 hours is likely to occur during head and neck free-flap surgery.^[14]^ In our case, the radial forearm free flap was selected as an axial pattern flap to save time because of the advantages of faster flap elevation and a more reliable vascular pedicle than other perforator flaps.

Intraoperatively, fluid administration and transfusions should be performed with caution. Maintaining a mean arterial pressure > 60 mm Hg should be targeted using goal-directed fluid therapy and vasopressors.^[13]^ Moreover, limiting intraoperative fluid therapy can reduce perfusion, hypotension, thrombosis, and adverse flap outcomes, whereas excessive fluid use can lead to tissue edema, dilutional coagulopathy, and eventually flap failure.^[5]^ Judicious fluid management that aims to maintain a urine output of 0.5 to 1 mL/kg/h and a normal level of dynamic indices, such as pulse pressure variation and serum lactate levels, can help guide fluid requirements. A hematocrit value of 30% can provide an optimum balance between oxygen delivery and blood viscosity.

One of the key goals during the postoperative phase is to minimize secondary ischemia arising from vessel spasms, intravascular thrombosis, and venous congestion secondary to hematoma or interstitial edema.^[6]^ Regular flap monitoring based on clinical observation is considered the “‘gold standard.’” However, other adjuncts, such as Doppler, implantable Doppler, microdialysis, fluorescence angiography, and near-infrared spectroscopy are also useful postoperatively. The use of vasoactive drugs is an important aspect of its management. Patients who are at a high risk of developing thrombosis due to various underlying conditions require administration of prophylactic perioperative unfractionated heparin (5000 units subcutaneously), which inhibits thrombin aggregation. Chemoprophylaxis with postoperative subcutaneous enoxaparin or unfractionated heparin is required for at least 14 days when the bleeding risk is considered minimal. Tranexamic acid is controversial but is worth considering.^[12,14]^

Free flap reconstruction should be considered a viable option for managing wound complications in patients with advanced lung cancer and brain metastases. Although advanced cancer poses certain challenges, including hypercoagulability and general anesthesia risks, a comprehensive evaluation of individual patients can help identify suitable candidates for this reconstructive technique. Considering the limited survival expectancy associated with advanced lung cancer and brain metastasis, careful discussion of the potential benefits and risks of surgery with patients and their families is crucial. Shared decision-making, consideration of the patient’s wishes, quality of life, and overall treatment goals, should guide the management plan.

## Author contributions

**Conceptualization:** Jung Soo Yoon.

**Data curation:** Dong Yun Lee, Jung Soo Yoon.

**Investigation:** Dong Yun Lee, Jung Soo Yoon.

**Supervision:** SooA Lim, SuRak Eo, Jung Soo Yoon.

**Writing – original draft:** Dong Yun Lee.

**Writing – review & editing:** Dong Yun Lee, SooA Lim, SuRak Eo, Jung Soo Yoon.
